# Efficacy and safety of local–regional therapy combined with chemotherapy, immune checkpoint inhibitors and lenvatinib as first-line treatment in advanced intrahepatic cholangiocarcinoma: a multicenter retrospective cohort study

**DOI:** 10.1007/s00262-025-04085-1

**Published:** 2025-05-30

**Authors:** Shuofeng Li, Guanhua Yu, Mingming Wang, Shi Feng, Shanshan Wang, Mingjian Piao, Chengjie Li, Zixiang Zhou, Ziyu Xun, Boyu Sun, Jiongyuan Li, Nan Zhang, Hu Li, Xiaobo Yang, Zhenyu Zhu, Haitao Zhao

**Affiliations:** 1 Department of Liver Surgery, Peking Union Medical College Hospital, Chinese Academy of Medical Sciences & Peking Union Medical College, Beijing, China; 2 Department of Hepatobiliary Surgery, The Fifth Medical Center of PLA General Hospital, Beijing, China

**Keywords:** Intrahepatic cholangiocarcinoma, Local–regional therapy, Chemotherapy, Immunotherapy, Targeted therapy

## Abstract

**Background:**

Local–regional therapy combined with immune checkpoint inhibitors (ICIs) and lenvatinib has shown promising anti-tumor activity in advanced biliary tract cancer. However, the efficacy and safety of integrating local–regional therapy with chemotherapy, ICIs, and lenvatinib in advanced intrahepatic cholangiocarcinoma (ICC) remain unclear. This study evaluated the efficacy and safety of first-line treatment combining local–regional therapy, chemotherapy, ICIs, and lenvatinib in advanced ICC.

**Methods:**

This multicenter study included 47 advanced ICC patients receiving local–regional therapy (radiotherapy, hepatic arterial infusion chemotherapy, or transarterial chemoembolization) plus chemotherapy, ICIs, and lenvatinib from October 2019 to January 2025. Outcomes included overall survival (OS), progression-free survival (PFS), objective response rate (ORR), disease control rate (DCR), adverse events (AEs), and prognostic factors analysis.

**Results:**

The multimodal therapy demonstrated mPFS of 10.2 months and mOS of 20.2 months. ORR and DCR reached 61.7% and 93.6%, respectively. Conversion surgery was performed in 10.6% (5/47) of patients, with 60.0% (3/5) achieving sustained remission. All patients experienced AEs, with grade 3–4 AEs in 66.0%, primarily including myelosuppression (23.4%), AST or ALT increased (19.1%), fatigue (14.9%), and pain (10.6%). No grade 5 AEs were observed, and all toxicities were manageable. Survival outcomes, tumor response rates, and grade 3–4 AE incidence showed no significant differences among local–regional therapy subgroups. Multivariate analyses identified impaired performance status as an independent predictor of poorer OS.

**Conclusions:**

The combined regimen of local–regional therapy, chemotherapy, ICIs, and lenvatinib exhibited marked efficacy and a tolerable safety profile, establishing it as a viable first-line approach for advanced ICC.

**Supplementary Information:**

The online version contains supplementary material available at 10.1007/s00262-025-04085-1.

## Introduction

Bile tract cancer (BTC), encompassing intrahepatic cholangiocarcinoma (ICC), extrahepatic cholangiocarcinoma, and gallbladder cancer, is a rare but highly aggressive malignancy [1]. ICC accounts for approximately 20% of BTC cases, with its incidence and mortality rates increasing in recent years [2–5]. Currently, chemotherapy-based regimens represent the mainstay treatments for advanced BTC, including ICC. Gemcitabine combined with cisplatin (GC chemotherapy) is recommended as first-line treatment for advanced ICC, whereas fluorouracil in combination with oxaliplatin and leucovorin calcium (FOLFOX chemotherapy) is the second-line treatment upon disease progression [6, 7]. Nevertheless, first-line and second-line chemotherapy regimens exhibited limited survival benefits with median overall survival (mOS) of under 1 year, underscoring the urgent need for novel therapeutic strategies demonstrating enhanced clinical outcomes.

Accumulating evidence demonstrates enhanced anti-tumor immune responses via inhibition of the PD-1/PD-L1 axis, positioning immune checkpoint inhibitors (ICIs) as promising agents for improving survival outcomes [8, 9]. Building on this rationale, multiple phase III trials have assessed immunotherapy–chemotherapy combinations in advanced BTC. In 2022, TOPAZ-1 study revealed that durvalumab (a PD-L1 inhibitor) plus GC chemotherapy achieved superior clinical outcomes in advanced BTC, reporting a progression-free survival (mPFS) of 7.2 months and mOS of 12.8 months [10]. In 2023, the KEYNOTE-966 trial similarly showed that pembrolizumab (a PD-1 inhibitor) plus GC chemotherapy significantly improved mPFS (6.5 months) and mOS (12.7 months) [11]. Furthermore, a phase II clinical trial conducted by Zhou Jian’s team revealed that combining ICIs with gemcitabine and oxaliplatin (GEMOX chemotherapy) and lenvatinib further improved the prognosis of advanced BTC patients, with mOS of 22.5 months and mPFS of 10.2 months [12]. Emerging real-world evidence have validated the clinical benefits of combining chemotherapy, ICIs, and lenvatinib. Reported mPFS extended from 8.6 to 13.7 months, with mOS spanning 13.4 to 23.8 months, markedly exceeding outcomes observed in advanced BTC patients receiving first-line ICIs–lenvatinib–chemotherapy combinations [13–15].

Local–regional therapies, including radiotherapy (RT), hepatic arterial infusion chemotherapy (HAIC), and transarterial chemoembolization (TACE), enhances the endogenous immune response by destroying tumor cells and releasing tumor-associated antigens, thereby generating synergistic effects with immunotherapy [16]. Previous studies showed that local–regional therapy combined with ICIs provides survival benefits for BTC patients [17–21]. A real-world study involving 49 advanced BTC patients found that local–regional therapy concurrent with lenvatinib and ICIs prolonged mPFS (7.9 months vs. 5.6 months) and mOS (13.7 months vs. 11.1 months), improving treatment response (ORR: 32% vs. 25%) compared to lenvatinib and ICIs alone [22]. Considering the promising efficacy of triple therapy combining chemotherapy, ICIs, and lenvatinib in advanced ICC, the addition of local–regional therapy may further enhance therapeutic outcomes. Currently, there is no research investigating the effectiveness and safety of combining local–regional therapy with chemotherapy, ICIs, and lenvatinib for advanced ICC.

Given the distinct anti-tumor mechanisms underlying local–regional therapy, ICIs, chemotherapy, and lenvatinib, integrating these approaches could generate synergistic interactions and enhance survival outcomes for advanced ICC patients. This multicenter study aimed to assess the clinical effectiveness and safety of first-line therapy that combines local–regional treatment with chemotherapy, ICIs, and lenvatinib for advanced ICC.

## Materials and methods

### Study population

This multicenter retrospective study included advanced ICC patients from Peking Union Medical College Hospital (PUMCH) and the Fifth Medical Center of the PLA General Hospital (PLAGH), who received first-line therapy with local–regional therapy combined with chemotherapy, ICIs, and lenvatinib between October 2019 and January 2025. The inclusion criteria were as follows: (1) histologically confirmed locally advanced or metastatic ICC; (2) age ≥ 18 years; (3) Eastern Cooperative Oncology Group (ECOG) performance status score of 0–1; (4) receiving at least two cycles of first-line chemotherapy combined with ICIs and lenvatinib, alongside concurrent local–regional treatments such as RT, HAIC, or TACE; and (5) having at least one tumor lesion assessable according to the Lesion per Response Evaluation Criteria in Solid Tumors (RECIST, version 1.1) [23]. Ultimately, a total of 47 advanced ICC patients were included. This study was carried out in full compliance with the Declaration of Helsinki and received approval from the Ethics Committees.

### Treatment protocol

The treatment start and end dates, drug dosages, cycle counts, and adverse events (AEs) were thoroughly documented for all patients enrolled in the study. All patients received systemic therapy comprising ≥ 2 cycles of GEMOX chemotherapy combined with ICIs and lenvatinib, alongside concurrent local–regional therapies. In GEMOX chemotherapy, gemcitabine (1000 mg/m^2^) was administered intravenously on days 1 and 8, and oxaliplatin (100 mg/m^2^) was administered intravenously on day 1, in 3 weeks cycles. ICIs were administered intravenously every three weeks, with dosages and dosing regimens adjusted to the specific drug. Lenvatinib was orally administered daily following a weight-stratified dosing protocol, with 12 mg prescribed for patients above 60 kg body weight and 8 mg for those not exceeding 60 kg.

Local–regional treatments included RT, HAIC, and TACE. The specific local–regional treatment plan was selected by the physician according to the tumor lesions, physical condition, and patient preferences. Local–regional therapy was administered either concomitantly with systemic therapy or within six weeks of its initiation. For RT, ICC patients received intensity-modulated radiation therapy (IMRT). The radiation dose, determined at the physician’s discretion, ranged from 24.0 to 60.0 Gy and was delivered in 6 to 27 fractions, with fraction doses ranging from 1.8 to 6.0 Gy, administered at a frequency of no more than five treatments per week. The treatment regimen was appropriately adjusted based on the individual condition to optimize therapeutic efficacy. For ICC patients presenting with intrahepatic lesions with rich blood supply, TACE and HAIC were recommended. In TACE, iodine oil (3–5 ml), fluorouracil (750 mg/m^2^), and oxaliplatin (60 mg/m^2^) were injected through the hepatic artery, with the chemotherapeutic agents infused over at least 15 min. In HAIC therapy, a microcatheter was introduced into the hepatic artery for drug infusion. The regimen consisted of oxaliplatin (40 mg/m^2^ over 2 h) and fluorouracil (800 mg/m^2^ over 22 h), administered every 3 to 4 weeks for 3 days, with folinic acid dripped intravenously (200 mg/m^2^ over 2 h) during fluorouracil infusion.

### Outcome assessment and safety evaluation

Imaging tests, including CT and MRI, were used to assess patient responses to treatment. The imaging examination was performed before the start of treatment and repeated every 6–8 weeks after each cycle of treatment. Imaging specialists from multicenters reviewed the CT and MRI results to determine the treatment response according to the RECIST v1.1 criteria. Treatment efficacy was evaluated using the objective response rate (ORR), which includes complete response (CR) and partial response (PR), along with the disease control rate (DCR) that incorporates CR, PR, and stable disease (SD). Other study endpoints included OS, PFS, and safety. OS was measured from treatment initiation to all-cause mortality, while PFS encompassed the period from first therapeutic intervention to radiologically confirmed disease progression or death. All AEs that occurred during treatment were recorded and classified using electronic medical records or follow-up surveys, and were assessed based on the Common Terminology Criteria for Adverse Events (CTCAE, version 5.0) [24].

### Statistical analysis

Data were analyzed as of February 15, 2025. Survival outcomes were analyzed using Kaplan–Meier methodology with log-rank testing for intergroup OS/PFS comparisons. Continuous and categorical variables were analyzed using t-test/Mann–Whitney U-test and Chi-square/Fisher’s exact test, respectively. Prognostic analysis via univariable and multivariable Cox regression identified independent clinicopathological determinants of OS and PFS, generating hazard ratios (HRs) with 95% CIs. Statistical significance adhered to a two-tailed P < 0.05 threshold, with R 4.2.0 executing all computations.

## Results

### Baseline characteristics of patients

**Fig. 1:**
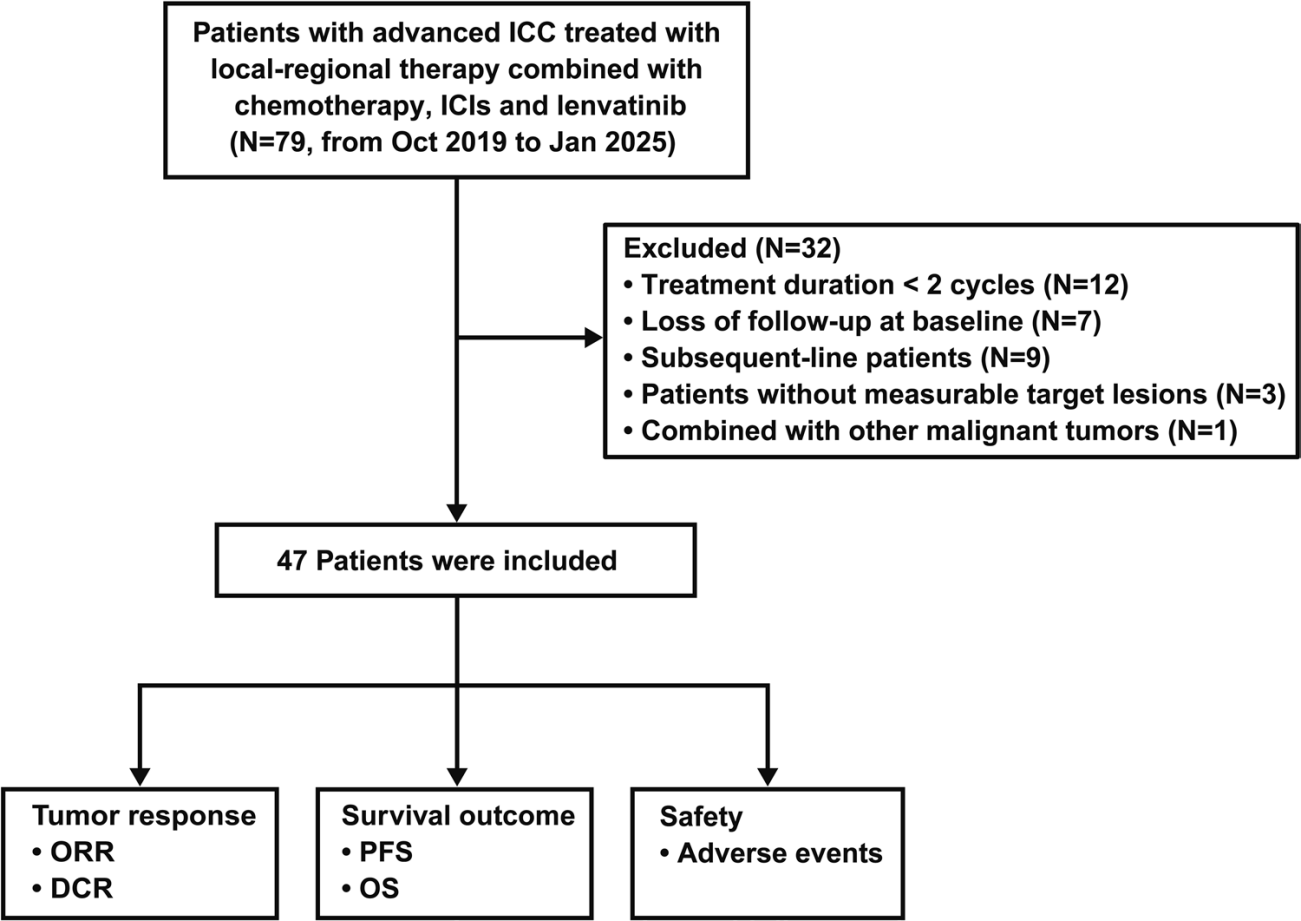
Study Flow Diagram. *ICC* intrahepatic cholangiocarcinoma, *ORR* objective response rate, *DCR* disease control rate, *PFS* progression-free survival, *OS* overall survival

A total of 79 patients with advanced ICC were initially enrolled. After excluding 32 patients, 47 were included in the study (Fig. 1). The baseline clinicopathologic characteristics were detailed in Table 1. 14 (29.8%) patients aged 60 or above, and 41 (87.2%) patients had an ECOG score of 0. In addition, 43 (91.5%) and 16 (34.0%) patients had Child–Pugh score A and positive HBV infection, respectively. Baseline CA19-9 ≥ 200 U/mL and CEA ≥ 5 U/mL were present in 19 (40.4%) and 15 (31.9%) patients, respectively. Pathological examination revealed that 29 (61.7%) patients had poorly differentiated tumors. Preoperative examination showed that 19 (40.4%) patients had locally advanced ICC, with the remaining 28 (59.6%) having metastatic ICC. Lymph nodes were the most frequent site of metastasis (37/47, 78.7%), followed by the liver (31/47, 66.0%), lungs (10/47, 21.3%), and bone (10/47, 21.3%). 18 (38.3%) patients received RT, 12 (25.5%) received HAIC, and the remaining 17 (36.2%) received TACE. Demographic and baseline characteristics of different local-regional therapy cohorts were detailed in **Supplementary Table 1‌**.

**Table 1:** Demographic and baseline characteristics of the entire cohort

Characteristic	Entire cohort (*n* = 47)
**Age, years**	
< 60	33 (70.2%)
≥ 60	14 (29.8%)
**Sex**	
Male	29 (61.7%)
Female	18 (38.3%)
**ECOG PS**	
0	41 (87.2%)
1	6 (12.8%)
**Child–Pugh score**	
A	43 (91.5%)
B	4 (8.5%)
**CA19-9**	
< 200 U/mL	28 (59.6%)
≥ 200 U/mL	19 (40.4%)
**CEA**	
< 5 U/mL	32 (68.1%)
≥ 5 U/mL	15 (31.9%)
**HBV infection**	
No	31 (66.0%)
Yes	16 (34.0%)
**Pathological grade**	
Median-high	18 (38.3%)
Low	29 (61.7%)
**Disease status**	
Locally advanced	19 (40.4%)
Metastatic	28 (59.6%)
**Lymph metastases**	
No	10 (21.3%)
Yes	37 (78.7%)
**Intrahepatic metastases**	
No	16 (34.0%)
Yes	31 (66.0%)
**Lung metastases**	
No	37 (78.7%)
Yes	10 (21.3%)
**Bone metastases**	
No	37 (78.7%)
Yes	10 (21.3%)
**Local regional therapy**	
RT	18 (38.3%)
TACE	17 (36.2%)
HAIC	12 (25.5%)

*RT* radiotherapy, *TACE* transarterial chemoembolization, *HAIC* hepatic arterial infusion chemotherapy

### Treatment and efficacy

As of February 15, 2025, all included patients underwent complete imaging evaluations. Local–regional therapy combined with chemotherapy, ICIs, and lenvatinib demonstrated promising treatment responses (Table 2). 3 (6.4%) patients achieved CR, 26 (55.3%) patients achieved PR, 15 (31.9%) patients had SD, and 3 (6.4%) patients had progressive disease (PD). The ORR reached an encouraging 61.7%, while the DCR was 93.6%. For patients receiving different local therapies, treatment responses were further analyzed. No statistically significant disparities in ORR (*P* = 0.184) or DCR (*P* = 1) were observed between the RT, TACE, and HAIC cohorts. Specifically, the RT cohort had an ORR of 61.1% and DCR of 94.4%, the TACE cohort had an ORR of 76.5% and DCR of 94.1%, and the HAIC cohort exhibited an ORR of 41.7% and DCR of 91.7%.

**Table 2:** Therapeutic efficacy of response and survival outcomes in the entire cohort, RT cohort, TACE cohort, and HAIC cohort

Therapeutic response assessment	Entire cohort (*n* = 47)	RT cohort (*n* = 18)	TACE cohort (*n* = 17)	HAIC cohort (*n* = 12)	*P* value
Complete response (CR, n, %)	3 (6.4%)	2 (11.1%)	0 (0.0%)	1 (8.3%)	–
Partial response (PR, n, %)	26 (55.3%)	9 (50.0%)	13 (76.5%)	4 (33.3%)	–
Stable disease (SD, n, %)	15 (31.9%)	6 (33.3%)	3 (17.6%)	6 (50.0%)	–
Progressive disease (PD, n, %)	3 (6.4%)	1 (5.6%)	1 (5.9%)	1 (8.3%)	–
Objective response rate (ORR, n, %)	29 (61.7%)	11 (61.1%)	13 (76.5%)	5 (41.7%)	0.184
Disease control rate (DCR, n, %)	44 (93.6%)	17 (94.4%)	16 (94.1%)	11 (91.7%)	1
Median progression-free survival (mPFS, months)	10.2	10.2	13.7	9.6	–
Median overall survival (mOS, months)	20.2	17.2	21.4	20.4	–

*RT* radiotherapy, *TACE* transarterial chemoembolization, *HAIC* hepatic arterial infusion chemotherapy

**Fig. 2:**
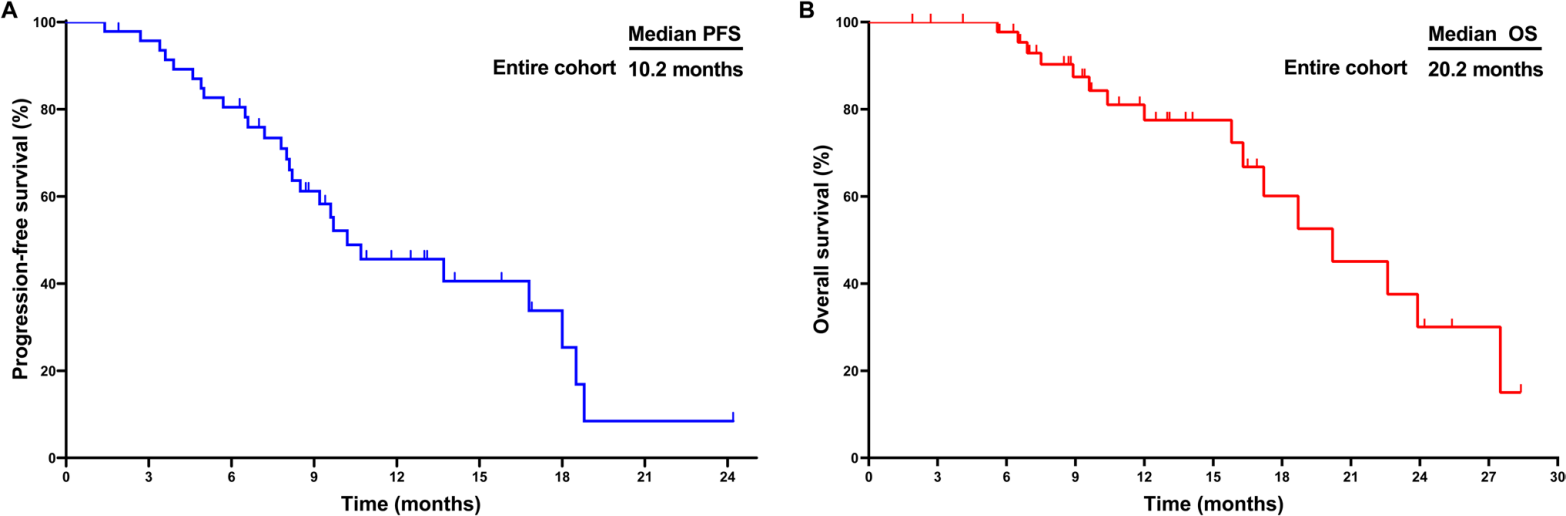
Kaplan–Meier Curves for PFS **A** and OS **B** in the entire cohort. *PFS* progression-free survival, *OS* overall survival

**Fig. 3:**
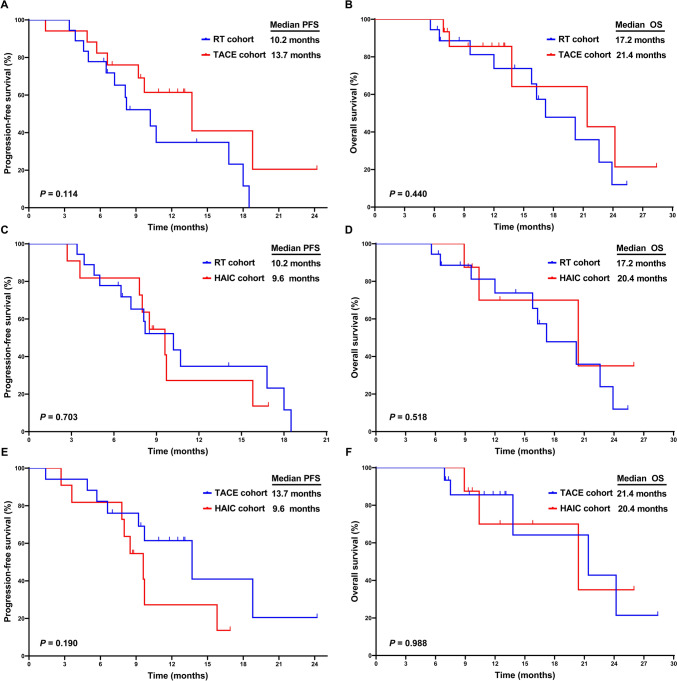
Kaplan–Meier Curves for PFS and OS in the RT cohort, TACE cohort and HAIC cohort. *RT *radiotherapy; *TACE* transarterial chemoembolization; *HAIC* hepatic arterial infusion chemotherapy, *PFS* progression-free survival, *OS* overall survival

At the prespecified data cutoff, mortality was observed in 38.3% (18/47) of the cohort. The mPFS and mOS were 10.2 and 20.2 months, respectively (Fig. 2). Prognosis showed no significant differences across RT, TACE, and HAIC cohorts (Fig. 3). The RT cohort showed a mPFS of 10.2 months and mOS of 17.2 months, while the TACE cohort exhibited a mPFS of 13.7 months and mOS of 21.4 months. For the HAIC cohort, mPFS and mOS were 9.6 months and 20.4 months, respectively. Notably, 5 of 47 patients (10.6%) patients underwent conversion surgery after receiving local–regional therapy combined with chemotherapy, ICIs, and lenvatinib as first-line treatment. All 5 patients achieved PR before the conversion surgery. These patients are still alive to date, with three of them remaining free of metastasis or recurrence at the final follow-up of this study. Clinical evidence suggested that local–regional therapy combined with chemotherapy, ICIs, and lenvatinib exhibited superior efficacy and promising survival outcomes.

**Fig. 4:**
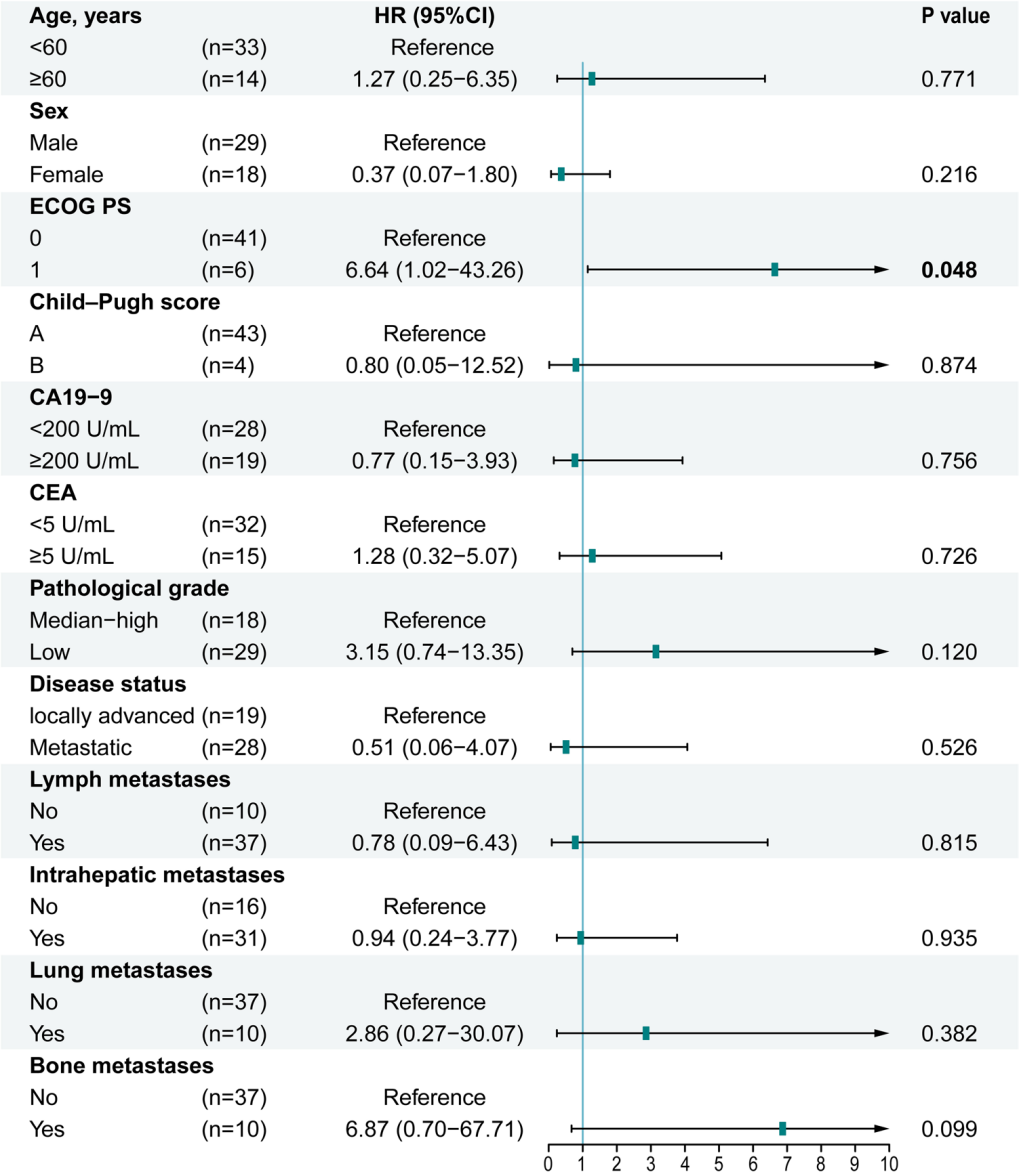
Forest plot of subgroup analysis for OS in the entire cohort. *OS* overall survival

Univariate and multivariate Cox models assessed prognostic factors for PFS and OS. Variables included age, sex, ECOG PS, Child–Pugh score, CA19-9, CEA, pathological grade, disease status, and metastatic sites, all analyzed multivariately (**Supplementary Table 2**). The results demonstrated that impaired ECOG PS (1 vs. 0; HR: 6.64; 95% CI: 1.02–43.26; *P* = 0.048) was associated with worse OS (Fig. 4).

### Safety

All patients in this study experienced AEs, but no grade 5 AEs were reported. The details of the AEs and their frequencies were shown in Table 3. Fatigue (23/47, 48.9%) was the most common AE, followed by myelosuppression (21/47, 44.7%), vomiting (20/47, 42.6%), pain (19/47, 40.4%), and hypertension (19/47, 40.4%). Grade 3–4 AEs occurred in 66.0% (31/47) of patients, primarily including myelosuppression (11/47, 23.4%), AST/ALT elevation (9/47, 19.1%), fatigue (7/47, 14.9%), and pain (5/47, 10.6%). Notably, all the AEs were tolerable and were reversible with active intervention, and no patient died or had all treatments completely interrupted due to AEs. For the most frequent grade 3–4 AEs observed in this study, specifically myelosuppression and AST/ALT elevation, interventions were implemented according to severity, primarily involving leukopoietic agents, hepatoprotective medications, dose modifications, or regimen adjustments when necessary. The specific management strategies for all AEs in this study were determined by clinicians based on a comprehensive evaluation of the patient’s physiological status, AE severity, and treatment response. Regarding different local treatment modalities, the incidence of grade 3–4 AEs was 50.0% in patients receiving RT, 83.3% in those receiving HAIC, and 70.6% in those receiving TACE (**Supplementary Table 3**). No statistically significant differences were found in the occurrence of grade 3–4 AEs across the groups that received different local–regional therapies (*P* = 0.178).

**Table 3:** Summary of commonly observed adverse events in the entire cohort

Events, n (%)	Entire cohort (*n* = 47)	
	Any grade	Grade 3–4
Total	47 (100.0%)	31 (66.0%)
Hypertension	19 (40.4%)	4 (8.5%)
Hand–foot syndrome	4 (8.5%)	0 (0.0%)
Rash	12 (25.5%)	3 (6.4%)
Hypothyroidism	4 (8.5%)	0 (0.0%)
Myelosuppression	21 (44.7%)	11 (23.4%)
Pruritus	4 (8.5%)	0 (0.0%)
Anemia	10 (21.3%)	0 (0.0%)
Proteinuria	3 (6.4%)	0 (0.0%)
Nausea	16 (34.0%)	1 (2.1%)
Vomiting	20 (42.6%)	1 (2.1%)
Decreased appetite	15 (31.9%)	0 (0.0%)
Decreased weight	2 (4.3%)	0 (0.0%)
Diarrhea	10 (21.3%)	1 (2.1%)
Abdominal distension	8 (17.0%)	0 (0.0%)
Constipation	4 (8.5%)	0 (0.0%)
Fatigue	23 (48.9%)	7 (14.9%)
Pain	19 (40.4%)	5 (10.6%)
Pneumonia	1 (2.1%)	1 (2.1%)
Oral ulcer	2 (4.3%)	0 (0.0%)
Dysphonia	2 (4.3%)	0 (0.0%)
Fever	14 (29.8%)	2 (4.3%)
Sore throat	2 (4.3%)	0 (0.0%)
Xerostomia	2 (4.3%)	0 (0.0%)
Cough	2 (4.3%)	1 (2.1%)
Total bilirubin increased	16 (34.0%)	4 (8.5%)
AST or ALT increased	18 (38.3%)	9 (19.1%)
Ascites	1 (2.1%)	1 (2.1%)
Hepatic encephalopathy	1 (2.1%)	0 (0.0%)

## Discussion

Compared to previous studies, this study was the first multicenter retrospective study to assess the effectiveness and safety of combining local–regional therapy with ICIs, chemotherapy, and targeted therapy for advanced ICC. This regimen exhibited promising efficacy, with a mPFS of 10.2 months and mOS of 20.2 months. Although AEs occurred in all patients, they remained manageable and reversible. These findings indicate that combining local–regional therapy with ICIs, chemotherapy, and lenvatinib represents a viable first-line strategy for advanced ICC.

ICC remains a highly aggressive malignancy characterized by unfavorable clinical outcomes. Emerging evidence from ICI studies has underscored the therapeutic potential of combining ICIs with chemotherapy, targeted agents, and local–regional modalities in advanced ICC [25–29]. Although ICIs combined with chemotherapy are currently endorsed as first-line therapy for advanced BTC, their limited clinical benefits highlight the pressing necessity for novel and optimized treatment approaches. Lenvatinib, a multi-target tyrosine kinase inhibitor, selectively inhibits vascular endothelial growth factor receptor (VEGFR) 1–3 and modulates the VEGF-VEGFR pathway to enhance anti-tumor immunity [30, 31]. Multiple studies highlighted the clinical benefits of ICIs combined with lenvatinib in advanced BTC [32–35]. A phase II trial (*n* = 38) in first-line advanced BTC demonstrated mOS of 17.7 months with this regimen [36]. Additionally, a single-arm study in advanced ICC validated the efficacy and safety of sindilizumab (a PD-1 inhibitor) plus lenvatinib as second-line therapy, achieving ORR of 46.3% and DCR of 70.3% [37]. Building on these findings, chemotherapy combined with ICIs and lenvatinib has emerged as a key focus in advanced ICC research. A single-arm phase II trial (*n* = 30) demonstrated that first-line triple therapy (ICIs, lenvatinib, chemotherapy) significantly improved outcomes, yielding mPFS of 10.2 months, mOS of 22.5 months, ORR of 80.0%, and DCR of 93.3% [12]. Real-world data from 57 advanced BTC patients further supported this regimen, showing mPFS of 9.27 months, mOS of 13.4 months, ORR of 43.9%, and DCR of 91.2% [14]. Collectively, these studies underscore the superior anti-tumor activity of chemotherapy combined with ICIs and lenvatinib over alternative regimens.

Local–regional therapies (RT, HAIC, and TACE) have shown potential in enhancing systemic treatment efficacy for advanced BTC. The integration of these modalities with systemic therapies offers a promising approach for advanced ICC [17–20, 29]. A retrospective study involving 49 advanced ICC patients demonstrated that first-line TACE combined with ICIs significantly extended mPFS (7.2 vs. 5.7 months) and mOS (13.2 vs. 7.6 months) compared to chemotherapy alone [38]. Additionally, advanced BTC patients treated with HAIC plus ICIs as subsequent-line therapy achieved a mOS of 8.8 months [21]. Further evidence indicates that combining local–regional therapy with ICIs and lenvatinib improves treatment responses and survival outcomes. For instance, advanced BTC patients receiving local–regional therapy alongside teraplizumab (a PD-1 inhibitor) and lenvatinib as subsequent-line therapy exhibited better ORR (32% vs. 25%), mPFS (7.9 vs. 5.6 months), and mOS (13.7 vs. 11.1 months) compared to teraplizumab concurrent with lenvatinib [22]. Similarly, RT combined with teraplizumab and lenvatinib improved mPFS (10.8 vs. 4.6 months) and mOS (13.7 vs. 9.2 months) [29]. Despite these advances, no prior studies have explored the combination of local–regional therapy with chemotherapy, ICIs, and lenvatinib in advanced ICC. To our knowledge, this is the first study evaluating the efficacy and safety of this first-line combination therapy in advanced ICC. Our findings revealed that adding local–regional therapy to chemotherapy, ICIs, and lenvatinib significantly enhanced prognosis, with mPFS of 10.2 months and mOS of 20.2 months. Notably, ORR (61.7%) and DCR (93.6%) surpassed those reported in earlier studies, suggesting synergistic interactions between local–regional and systemic therapies to amplify ICIs–chemotherapy–lenvatinib efficacy in advanced ICC.

The rationale underlying this multimodal therapeutic approach may stem from the synergistic interaction between locoregional interventions and systemic treatments. Radiotherapy induces direct cytotoxic damage while simultaneously triggering tumor antigen release to mobilize systemic immune responses, facilitating CD8^+^ T cell infiltration and activation while diminishing immunosuppressive T cell populations, thereby mitigating immune tolerance and sensitizing tumors to ICIs therapy [39–41]. This modality additionally amplifies proinflammatory cytokine secretion (notably IL-6 and TNF-α) that cooperates with ICIs to intensify T cell-dependent anti-tumor activity [40, 41]. HAIC achieves superior intratumoral drug concentrations through hepatic arterial infusion, with its sustained delivery profile potentially reactivating adaptive immunity and restoring immune surveillance mechanisms to potentiate immunotherapy [42]. TACE generates mechanical obstruction of tumor vasculature, combined with lenvatinib-induced VEGF signaling blockade that collectively inhibits neoplastic angiogenesis [43, 44]. These antiangiogenic actions not only compromise tumor nutrient provision but also remodel the immune microenvironment through CD8^+^ T cell proportion elevation, regulatory T cell population reduction, and suppression of immunosuppressive mediators including TGF-β and IL-10, collectively optimizing ICIs efficacy [45]. Further mechanistic exploration is essential to refine the combination of modalities, dosing schedules, and intervention timing for this multimodal regimen.

Beyond its clinical efficacy, the combination of local–regional therapy with chemotherapy, ICIs, and lenvatinib exhibited a manageable safety profile in this study. Previous trials evaluating first-line chemotherapy combined with ICIs and lenvatinib in advanced BTC reported grade 3–4 AE frequencies ranging from 41.5 to 56.7% [12–14]. In our cohort, the addition of local–regional therapy did not exacerbate toxicity, with grade 3–4 AEs observed in 66.0% of patients, consistent with prior reports on chemotherapy plus ICIs and lenvatinib. Myelosuppression remained the most frequent grade 3–4 AE, followed by elevated AST/ALT, fatigue, and pain, aligning with severe AE patterns in earlier studies [12–15]. Importantly, while all patients experienced AEs of varying severity, none required treatment discontinuation or experienced fatal events, and all toxicities were controllable and reversible with prompt interventions. These results affirm that combining local–regional therapy with chemotherapy, ICIs, and lenvatinib maintains an acceptable safety profile, supporting its feasibility as a first-line approach for advanced ICC patients with a favorable performance status (ECOG PS 0/1).

This study has several limitations. First, the sample size remains relatively limited, potentially attributable to the inherent rarity of ICC which constitutes less than 1% of all human malignancies [46]. Furthermore, while current clinical guidelines recommend chemotherapy with or without ICIs as first-line treatment for advanced ICC, the multimodal regimen integrating local–regional therapy with ICIs, chemotherapy, and lenvatinib has not yet been adopted into standard treatment protocols, limiting patient access to this multimodal approach [47]. Second, the absence of a control group receiving ICIs combined with chemotherapy and lenvatinib limits the ability to definitively evaluate the additional benefits of local–regional therapy in this regimen. Although a previous study involving 53 advanced ICC patients treated with first-line ICIs, chemotherapy, and lenvatinib reported mPFS and mOS of 8.63 and 14.3 months, respectively, the inclusion criteria and baseline characteristics of that cohort closely matched those of our study, suggesting potential comparability [13]. The incorporation of local–regional therapy into this triple regimen extended mPFS and mOS by 1.57 and 5.9 months, respectively, indicating potential synergistic effects through combined modalities. Furthermore, compared to guideline-recommended standard therapies for advanced BTC (chemotherapy with or without ICIs; mPFS 5.6–8.0 months, mOS 11.7–12.8 months), our novel regimen demonstrated superior mPFS (10.2 months) and mOS (20.2 months), suggesting substantial survival advantages over conventional treatments [6, 10, 11]. Nevertheless, future clinical studies incorporating expanded patient cohorts and control groups remain imperative to corroborate the conclusions of this investigation. Third, as a retrospective cohort study, potential biases arising from non-randomized design and incomplete data entries may affect result reliability. Thus, prospective multicenter trials are essential to confirm the efficacy and safety of local–regional therapy concurrent with chemotherapy, ICIs, and lenvatinib in advanced ICC. Further research should also explore optimal timing and dosing of local–regional therapy to maximize synergistic benefits. Nonetheless, as the first investigation evaluating this multimodal approach in advanced ICC, this investigation offers critical preliminary evidence to guide first-line regimen selection and inform future trial designs.

## Conclusion

The integration of local–regional therapy with chemotherapy, ICIs, and lenvatinib may amplify therapeutic efficacy and extend survival in advanced ICC patients, while preserving a manageable safety profile. This multimodal regimen emerges as a viable and tolerable first-line strategy for advanced ICC. Further validation through prospective trials is essential to confirm these outcomes.

## Supplementary Information

Below is the link to the electronic supplementary material.

Supplementary file1 (DOCX 26 KB)

## Data Availability

All data supporting the results of the study are available upon reasonable request.
