# Supplementary material for: Efficacy and safety of local–regional therapy combined with chemotherapy, immune checkpoint inhibitors and lenvatinib as first-line treatment in advanced intrahepatic cholangiocarcinoma: a multicenter retrospective cohort study
Source: Cancer Immunol Immunother. 2025 May 30;74(7):229. doi: 10.1007/s00262-025-04085-1 (PMC12125452; doi:10.1007/s00262-025-04085-1)
Supplement: Supplementary file 1 — Supplementary file1 (DOCX 26 KB) [file 262_2025_4085_MOESM1_ESM.docx]

**Supplementary Tables:**

**Supplementary Table 1. Demographic and baseline characteristics of different local-regional therapy cohorts**

| **Characteristic** | **RT cohort  (n=18)** | **TACE cohort (n=17)** | **HAIC cohort (n=12)** | **P-value** |
| --- | --- | --- | --- | --- |
| **Age, years** |  |  |  | 0.923 |
| <60 | 12 (66.7%) | 12 (70.6%) | 9 (75.0%) |  |
| ≥60 | 6 (33.3%) | 5 (29.4%) | 3 (25.0%) |  |
| **Sex** |  |  |  | 0.413 |
| Male | 9 (50.0%) | 12 (70.6%) | 8 (66.7%) |  |
| Female | 9 (50.0%) | 5 (29.4%) | 4 (33.3%) |  |
| **ECOG PS** |  |  |  | 0.119 |
| 0 | 15 (83.3%) | 17 (100.0%) | 9 (75.0%) |  |
| 1 | 3 (16.7%) | 0 (0.0%) | 3 (25.0%) |  |
| **Child–Pugh score** |  |  |  | 0.825 |
| A | 17 (94.4%) | 15 (88.2%) | 11 (91.7%) |  |
| B | 1 (5.6%) | 2 (11.8%) | 1 (8.3%) |  |
| **CA19-9** |  |  |  | 0.022 |
| <200 U/mL | 13 (72.2%) | 12 (70.6%) | 3 (25.0%) |  |
| ≥200 U/mL | 5 (27.8%) | 5 (29.4%) | 9 (75.0%) |  |
| **CEA** |  |  |  | 0.236 |
| <5 U/mL | 10 (55.6%) | 14 (82.4%) | 8 (66.7%) |  |
| ≥5 U/mL | 8 (44.4%) | 3 (17.6%) | 4 (33.3%) |  |
| **HBV infection** |  |  |  | 0.799 |
| No | 11 (61.1%) | 11 (64.7%) | 9 (75.0%) |  |
| Yes | 7 (38.9%) | 6 (35.3%) | 3 (25.0%) |  |
| **Pathological grade** |  |  |  | 0.122 |
| Median-high | 4 (22.2%) | 7 (41.2%) | 7 (58.3%) |  |
| Low | 14 (77.8%) | 10 (58.8%) | 5 (41.7%) |  |
| **Disease status** |  |  |  | 0.868 |
| Locally advanced | 8 (44.4%) | 7 (41.2%) | 4 (33.3%) |  |
| Metastatic | 10 (55.6%) | 10 (58.8%) | 8 (66.7%) |  |
| **Lymph metastasis** |  |  |  | 0.257 |
| No | 6 (33.3%) | 3 (17.6%) | 1 (8.3%) |  |
| Yes | 12 (66.7%) | 14 (82.4%) | 11 (91.7%) |  |
| **Intrahepatic metastasis** |  |  |  | 0.464 |
| No | 5 (27.8%) | 5 (29.4%) | 6 (50.0%) |  |
| Yes | 13 (72.2%) | 12 (70.6%) | 6 (50.0%) |  |
| **Lung metastasis** |  |  |  | 0.819 |
| No | 15 (83.3%) | 13 (76.5%) | 9 (75.0%) |  |
| Yes | 3 (16.7%) | 4 (23.5%) | 3 (25.0%) |  |
| **Bone metastasis** |  |  |  | 0.289 |
| No | 12 (66.7%) | 15 (88.2%) | 10 (83.3%) |  |
| Yes | 6 (33.3%) | 2 (11.8%) | 2 (16.7%) |  |

RT, radiotherapy; TACE, transarterial chemoembolization; HAIC, hepatic arterial infusion chemotherapy.

**Supplementary Table 2.** **Univariate and Multivariate Cox Regression Analysis of Prognostic Factors for PFS and OS.**

| **Characteristic** | **Univariates COX analysis for PFS** | | **Multivariates COX analysis for PFS** | | **Univariates COX analysis for OS** | | **Multivariates COX analysis for OS** | |
| --- | --- | --- | --- | --- | --- | --- | --- | --- |
|  | **HR (95%CI)** | **P-value** | **HR (95%CI)** | **P-value** | **HR (95%CI)** | **P-value** | **HR (95%CI)** | **P-value** |
| **Age, years** |  |  |  |  |  |  |  |  |
| <60 | Reference |  | Reference |  | Reference |  | Reference |  |
| ≥60 | 0.74 (0.32-1.67) | 0.463 | 0.79 (0.26-2.41) | 0.675 | 1.77 (0.64-4.93) | 0.275 | 1.27 (0.25-6.35) | 0.771 |
| **Sex** |  |  |  |  |  |  |  |  |
| Male | Reference |  | Reference |  | Reference |  | Reference |  |
| Female | 0.53 (0.23-1.21) | 0.134 | 0.42 (0.15- 1.15) | 0.092 | 0.70 (0.25-1.97) | 0.493 | 0.37 (0.07-1.80) | 0.216 |
| **ECOG PS** |  |  |  |  |  |  |  |  |
| 0 | Reference |  | Reference |  | Reference |  | Reference |  |
| 1 | 0.99 (0.37-2.69) | 0.988 | 1.45 (0.38-5.55) | 0.586 | 3.47 (1.25-9.65) | 0.017 | 6.64 (1.02-43.26) | 0.048 |
| **Child–Pugh score** |  |  |  |  |  |  |  |  |
| A | Reference |  | Reference |  | Reference |  | Reference |  |
| B | 0.90 (0.27- 3.03) | 0.862 | 0.59 (0.07-4.82) | 0.620 | 1.26 (0.36-4.41) | 0.713 | 0.80 (0.05-12.52) | 0.874 |
| **CA19-9** |  |  |  |  |  |  |  |  |
| <200 U/mL | Reference |  | Reference |  | Reference |  | Reference |  |
| ≥200 U/mL | 0.90 (0.40-2.01) | 0.889 | 0.80 (0.30-2.14) | 0.661 | 1.09 (0.42-2.83) | 0.855 | 0.77 (0.15-3.93) | 0.756 |
| **CEA** |  |  |  |  |  |  |  |  |
| <5 U/mL | Reference |  | Reference |  | Reference |  | Reference |  |
| ≥5 U/mL | 1.02 (0.47-2.23) | 0.955 | 1.11 (0.40-3.04) | 0.840 | 1.56 (0.61-3.98) | 0.349 | 1.28 (0.32-5.07) | 0.726 |
| **Pathological grade** |  |  |  |  |  |  |  |  |
| Median-High | Reference |  | Reference |  | Reference |  | Reference |  |
| Low | 1.29 (0.61-2.76) | 0.505 | 1.59 (0.60-4.17) | 0.349 | 3.09 (1.10-8.74) | 0.033 | 3.15 (0.74-13.35) | 0.120 |
| **Disease status** |  |  |  |  |  |  |  |  |
| locally advanced |  |  | Reference |  | Reference |  | Reference |  |
| Metastatic | 0.93 (0.45-1.94) | 0.842 | 0.46 (0.13-1.64) | 0.229 | 2.05 (0.73-5.76) | 0.175 | 0.51 (0.06-4.07) | 0.526 |
| **Lymph metastases** |  |  |  |  |  |  |  |  |
| No | Reference |  | Reference |  | Reference |  | Reference |  |
| Yes | 1.34 (0.54-3.33) | 0.529 | 0.92 (0.25-3.41) | 0.896 | 2.17 (0.63-7.53) | 0.222 | 0.78 (0.09-6.43) | 0.815 |
| **Intrahepatic metastases** |  |  |  |  |  |  |  |  |
| No | Reference |  | Reference |  | Reference |  | Reference |  |
| Yes | 0.79 (0.36-1.73) | 0.558 | 1.10 (0.39-3.15) | 0.854 | 1.02 (0.38-2.72) | 0.976 | 0.94 (0.24-3.77) | 0.935 |
| **Lung metastases** |  |  |  |  |  |  |  |  |
| No | Reference |  | Reference |  | Reference |  | Reference |  |
| Yes | 1.03 (0.44-2.44) | 0.942 | 2.01 (0.39-10.53) | 0.406 | 1.05 (0.39-2.86) | 0.923 | 2.86 (0.27-30.07) | 0.382 |
| **Bone metastases** |  |  |  |  |  |  |  |  |
| No | Reference |  | Reference |  | Reference |  | Reference |  |
| Yes | 1.37 (0.55-3.42) | 0.500 | 3.08 (0.67-14.20) | 0.149 | 1.88 (0.66-5.36) | 0.240 | 6.87 (0.70-67.71) | 0.099 |

RT, radiotherapy; TACE, transarterial chemoembolization; HAIC, hepatic arterial infusion chemotherapy; PFS, progression-free survival; OS, overall survival.

**Supplementary Table 3. Summary of commonly observed adverse events in different local-regional therapy cohorts.**

| **Events, n (%)** | **RT cohort**  **(n=18)** | | **TACE cohort**  **(n=17)** | | **HAIC cohort**  **(n=12)** | | **P-value** | **P-value** |
| --- | --- | --- | --- | --- | --- | --- | --- | --- |
|  | Any grade | Grade 3-4 | Any grade | Grade 3-4 | Any grade | Grade 3-4 | Any grade | Grade  3 -4 |
| **Total** | 18 (100.0%) | 9 (50.0%) | 17 (100.0%) | 12 (70.6%) | 12 (100.0%) | 10 (83.3%) | 1 | 0.178 |
| Hypertension | 5 (27.8%) | 0 (0.0%) | 8 (47.1%) | 3 (17.6%) | 6 (50.0%) | 1 (8.3%) | 0.393 | 0.122 |
| Hand-foot syndrome | 2 (11.1%) | 0 (0.0%) | 1 (5.9%) | 0 (0.0%) | 1 (8.3%) | 0 (0.0%) | 1 | - |
| Rash | 5 (27.8%) | 2 (11.1%) | 3 (17.6%) | 0 (0.0%) | 4 (33.3%) | 1 (8.3%) | 0.643 | 0.462 |
| Hypothyroidism | 2 (11.1%) | 0 (0.0%) | 2 (11.8%) | 0 (0.0%) | 0 (0.0%) | 0 (0.0%) | 0.660 | - |
| Myelosuppression | 7 (38.9%) | 3 (16.7%) | 8 (47.1%) | 4 (23.5%) | 6 (50.0%) | 4 (33.3%) | 0.810 | 0.563 |
| Pruritus | 2 (11.1%) | 0 (0.0%) | 1 (5.9%) | 0 (0.0%) | 1 (8.3%) | 0 (0.0%) | 1 | - |
| Anemia | 2 (11.1%) | 0 (0.0%) | 6 (35.3%) | 0 (0.0%) | 2 (16.7%) | 0 (0.0%) | 0.228 | - |
| Proteinuria | 1 (5.6%) | 0 (0.0%) | 2 (11.8%) | 0 (0.0%) | 0 (0.0%) | 0 (0.0%) | 0.613 | - |
| Nausea | 4 (22.2%) | 0 (0.0%) | 6 (35.3%) | 1 (5.9%) | 6 (50.0%) | 0 (0.0%) | 0.267 | 0.617 |
| Vomiting | 6 (33.3%) | 0 (0.0%) | 7 (41.2%) | 1 (5.9%) | 7 (58.3%) | 0 (0.0%) | 0.394 | 0.617 |
| Decreased appetite | 5 (27.8%) | 0 (0.0%) | 6 (35.3%) | 0 (0.0%) | 4 (33.3%) | 0 (0.0%) | 0.924 | - |
| Gastrointestinal hemorrhage | 0 (0.0%) | 0 (0.0%) | 0 (0.0%) | 0 (0.0%) | 0 (0.0%) | 0 (0.0%) | - | - |
| Decreased weight | 1 (5.6%) | 0 (0.0%) | 1 (5.9%) | 0 (0.0%) | 0 (0.0%) | 0 (0.0%) | 1 | - |
| Diarrhea | 1 (5.6%) | 0 (0.0%) | 5 (29.4%) | 1 (5.9%) | 4 (33.3%) | 0 (0.0%) | 0.120 | 0.617 |
| Abdominal distension | 5 (27.8%) | 0 (0.0%) | 2 (11.8%) | 0 (0.0%) | 1 (8.3%) | 0 (0.0%) | 0.416 | - |
| Constipation | 3 (16.7%) | 0 (0.0%) | 0 (0.0%) | 0 (0.0%) | 1 (8.3%) | 0 (0.0%) | 0.227 | - |
| Fatigue | 10 (55.6%) | 1 (5.6%) | 8 (47.1%) | 4 (23.5%) | 5 (41.7%) | 2 (16.7%) | 0.743 | 0.322 |
| Pain | 8 (44.4%) | 2 (11.1%) | 5 (29.4%) | 2 (11.8%) | 6 (50.0%) | 1 (8.3%) | 0.559 | 1 |
| Drowsiness | 0 (0.0%) | 0 (0.0%) | 0 (0.0%) | 0 (0.0%) | 0 (0.0%) | 0 (0.0%) | - | - |
| Insomnia | 0 (0.0%) | 0 (0.0%) | 0 (0.0%) | 0 (0.0%) | 0 (0.0%) | 0 (0.0%) | - | - |
| Pneumonia | 0 (0.0%) | 0 (0.0%) | 0 (0.0%) | 0 (0.0%) | 1 (8.3%) | 1 (8.3%) | 0.255 | 0.255 |
| Oral ulcer | 2 (11.1%) | 0 (0.0%) | 0 (0.0%) | 0 (0.0%) | 0 (0.0%) | 0 (0.0%) | 0.328 | - |
| Dysphonia | 0 (0.0%) | 0 (0.0%) | 1 (5.9%) | 0 (0.0%) | 1 (8.3%) | 0 (0.0%) | 0.517 | - |
| Fever | 2 (11.1%) | 0 (0.0%) | 9 (52.9%) | 1 (5.9%) | 3 (25.0%) | 1 (8.3%) | 0.027 | 0.517 |
| Sore throat | 1 (5.6%) | 0 (0.0%) | 1 (5.9%) | 0 (0.0%) | 0 (0.0%) | 0 (0.0%) | 1 | - |
| Xerostomia | 2 (11.1%) | 0 (0.0%) | 0 (0.0%) | 0 (0.0%) | 0 (0.0%) | 0 (0.0%) | 0.328 | - |
| Cough | 1 (5.6%) | 1 (5.6%) | 1 (5.9%) | 0 (0.0%) | 0 (0.0%) | 0 (0.0%) | 1 | 1 |
| Total bilirubin increased | 4 (22.2%) | 1 (5.6%) | 7 (41.2%) | 0 (0.0%) | 5 (41.7%) | 3 (25.0%) | 0.431 | 0.076 |
| Hepatitis | 0 (0.0%) | 0 (0.0%) | 0 (0.0%) | 0 (0.0%) | 0 (0.0%) | 0 (0.0%) | - | - |
| AST or ALT increased | 6 (33.3%) | 5 (27.8%) | 5 (29.4%) | 1 (5.9%) | 7 (58.3%) | 3 (25.0%) | 0.266 | 0.249 |
| Ascites | 0 (0.0%) | 0 (0.0%) | 0 (0.0%) | 0 (0.0%) | 1 (8.3%) | 1 (8.3%) | 0.255 | 0.255 |
| Hepatic encephalopathy | 1 (5.6%) | 0 (0.0%) | 0 (0.0%) | 0 (0.0%) | 0 (0.0%) | 0 (0.0%) | 1 | - |

RT, radiotherapy; TACE, transarterial chemoembolization; HAIC, hepatic arterial infusion chemotherapy.
